# Nutrition transition among adolescents of a south-Mediterranean country: dietary patterns, association with socio-economic factors, overweight and blood pressure. A cross-sectional study in Tunisia

**DOI:** 10.1186/1475-2891-10-38

**Published:** 2011-04-24

**Authors:** Hajer Aounallah-Skhiri, Pierre Traissac, Jalila El Ati, Sabrina Eymard-Duvernay, Edwige Landais, Noureddine Achour, Francis Delpeuch, Habiba Ben Romdhane, Bernard Maire

**Affiliations:** 1 National Institute of Public Health (INSP), 5-7 rue Khartoum, Tunis, Tunisia; 2 Doctoral School 393, Université Pierre et Marie Curie, Paris, France; 3 IRD (Institut de Recherche pour le Développement), UMR 204 NUTRIPASS, IRD-UM1-UM2, 911, av. Agropolis, 34394 Montpellier, France; 4 National Institute of Nutrition and Food Technology (INNTA), Tunis, Tunisia

## Abstract

**Background:**

The increase in the burden of chronic diseases linked to the nutrition transition and associated dietary and lifestyle changes is of growing concern in south and east Mediterranean countries and adolescents are at the forefront of these changes. This study assessed dietary intake and association with socio-economic factors and health outcomes among adolescents in Tunisia.

**Methods:**

Cross-sectional survey (year 2005); 1019 subjects 15-19 y. from a clustered random sample. Dietary intake was assessed by a validated semi-quantitative frequency questionnaire (134 items) as was physical activity; the Diet Quality Index International measured diet quality; dietary patterns were derived by multiple correspondence analysis from intakes of 43 food groups. Body Mass Index (BMI) ≥85^th ^and 95^th ^percentile defined overweight and obesity. Waist Circumference (WC) assessed abdominal fat. High blood pressure was systolic (SBP) or diastolic blood pressure (DBP) ≥90^th ^of the international reference for 15-17 y., and SBP/DBP ≥120/80 mm Hg for 18-19 y.

**Results:**

Energy intake levels were quite high, especially for females. The macro-nutrient structure was close to recommendations but only 38% had a satisfactory diet quality. A main traditional to modern dietary gradient, linked to urbanisation and increased economic level, featured an increasing consumption of white bread, dairy products, sugars, added fats and fruits and decreasing consumption of oils, grains, legumes and vegetables; regarding nutrients this modern diet score featured a decreasing relationship with total fat and an increase of calcium intake, but with an increase of energy, sugars and saturated fat, while vitamin C, potassium and fibre decreased. Adjusted for age, energy and physical activity, this modern pattern was associated with increased overweight in males (2^nd ^vs. 1^st ^tertile: Prevalence Odds-Ratio (POR) = 4.0[1.7-9.3], 3^rd ^vs. 1^st^: POR = 3.3[1.3-8.7]) and a higher WC. Adjusting also for BMI and WC, among females, it was associated with decreased prevalence of high blood pressure (2^nd ^vs. 1^st ^tertile: POR = 0.5[0.3-0.8], 3^rd ^vs. 1^st ^tertile: POR = 0.4[0.2-0.8]).

**Conclusion:**

The dietary intake contrasts among Tunisian adolescents, linked to socio-economic differentials are characteristic of a nutrition transition situation. The observed gradient of modernisation of dietary intake features associations with several nutrients involving a higher risk of chronic diseases but might have not only negative characteristics regarding health outcomes.

## Background

The prevalence of overweight among children and adolescents is increasing worldwide [1-3] while the proportion of overweight adolescents who become obese in adulthood appears to be rather high [4]. During a period of their life that is characterised by important psychological and physiological changes, adolescents are frequently attracted to unhealthy lifestyles [5,6], and rarely prefer food with the best nutritional value [7,8]. Adolescents in developing countries also display an increasing incidence of overweight and obesity; often prompt to adopt the nutrition transition related-lifestyle [9], they are thus exposed much sooner - and will be exposed longer - than the preceding generation to health problems such as obesity, diabetes, cardiovascular risk factors and some cancers [1,4,6,10]. Emblematic of a number of similarly emerging south and east Mediterranean countries [11,12], Tunisia is also presently undergoing an active epidemiological and nutritional transition [13,14] with a rapid increase in adult and child overweight and in the prevalence of co-morbidities [15-17]. Also, due to the associated demographic transition, adolescents represent a growing proportion of the population of such emerging countries, e.g. at the time of the study in Tunisia 20.7% and 10.7% for the 10-19 and 15-19 y. age groups respectively [18]. Nevertheless, few studies are available yet regarding nutritional transition related dietary, socio-economic and health issues among youth in the Arab world, especially pertaining to south Mediterranean countries [19-21]: thus the objectives of the study were to describe the dietary intake of Tunisian adolescents from different perspectives such as foods, nutrients, diet quality as well as multivariate dietary patterns, and to assess the associations with socio-economic factors and health outcomes such as anthropometry and blood pressure (BP).

## Subjects and methods

### Design and sampling

#### Study area

Tunisia is a North African country with a population of about ten million [18], having recently undergone a steady and rapid economic development and currently featuring an upper middle level of development (ranked 89^th ^out of 177 on the Human Development Index composite scale in 2005 [22]). Geographical differences (inland along the Algerian border in the west, coastal along the Mediterranean Sea in the north and east), as well as a climatic and agricultural gradient (from Mediterranean in the north to arid in the south) result in marked development contrasts between the administrative regions of the country.

#### Design and sampling

The target population was all 15-19-year-old adolescents from three regions chosen to represent a diversity of food habits and nutritional status [23]: the urbanised and highly developed District of Tunis around the capital city; the tourism oriented, economically relatively well-developed coastal Middle Eastern region; and the more remote rural inland Middle Western region. The cross sectional survey was conducted from April to September 2005: in each of the three regions, 47 clusters (census districts from the 2004 national census) were selected with probability proportional to size, and then 20 households were selected at random within each district. All 15-19-year-old adolescents from the 2820 selected households were eligible.

### Measurements and derived variables

#### Environmental, socio-economic and physiological factors

A proxy for the economic level of the household was derived from multivariate analysis of items relevant in the Tunisian context, such as type of house, number of people per room, type of drinking water supply, type of sanitation, and possessions such as car, refrigerator, television, computer, satellite dish antenna [17,24]. The profession and education of the father and the mother were recorded. Information collected concerning the adolescent was sex, age and whether currently registered at school.

#### Physical activity

A validated one month retrospective frequency questionnaire, adapted from one previously used for Tunisian adults [25] was used to assess physical activity. The metabolic equivalent (MET) of daily activities was derived using an international compendium of physical activity [26]; activities were then classified as light, moderate or vigorous (respectively <3, 3-6 and ≥6 METs; 1 MET = 3.5 mL O²/kg) [27]. Variables pertaining to percentages of light, moderate and vigorous daytime activities of each subject were computed and coded into tertiles for specific analyses.

#### Dietary intake

One month retrospective food consumption was assessed using a validated semi-quantitative food frequency questionnaire (FFQ) adapted to the Tunisian context [28]: for 134 items (Table 1), subjects were asked to report both the frequency of consumption and the quantity using a combination of visual tools. A specific Tunisian food composition database [29] and the Food Processor software [30] were used to compute average daily intake of energy (kcal or kJ/day), and macro- and micro-nutrients. Energy in % of requirements [31] was computed using total energy expenditure assessed by the physical activity questionnaire. Extreme and likely inaccurate records (energy intake ≥ 95^th ^percentile of the observed distribution or less than the estimated basal metabolic rate) were excluded from the analyses [32,33]. The Diet Quality Index-International (DQI-I) was used to characterise diet quality: the global and detailed four categories of score (variety, adequacy, moderation, balance) were used as such as interval variables in analyses as well as global diet quality score in tertiles and a "good overall diet quality" (defined as a global score ≥60) binary variable [34].

**Table 1:** Food list (134 items) and 43 food groups used for computation of dietary patterns

Food Groups (43)	Food List (134 items of the retrospective food frequency questionnaire)
1-White bread	French bread
2-Milk	Whole milk, semi-skimmed milk, skimmed milk, condensed & sweetened milk, goat milk
3-Cheese	Semi-hard cheese, Emmental cheese, uncured cheese 30%
4-Yogurt	Semi-skimmed yogurt
5-Chamia^1^, nuts, chocolate	Chamia, almond nuts, sunflower seeds, sesame seeds, chocolate bar, hazelnut chocolate, milk chocolate, dried raisin.
6-Sugars	Jam, white sugar cubes, white sugar granulated powder, sweets
7-Sweetened beverages	Sodas
8-Butter	Butter
9-Lamb fat	Cooked lamb fat
10-Fruits and fresh fruit juices	Dates, orange, mandarin, grapefruit, pear, apple, fresh lemon juice, fresh orange juice
11-Processed fruits	Green olive in brine, ripe olive in brine
12-Eggs	Fresh egg, fried egg, boiled egg
13-Fish	Broiled mackerel, fried fish, grilled fish
14-Other sea food	Boiled cuttlefish, cooked dried octopus
15-Canned fish	Canned tuna
16-Lamb Meat	Boiled lamb meat, braised lamb meat, grilled lamb meat
17-Beef meat	Grilled beef meat, stew beef meat
18-Goat meat	Boiled goat meat
19-Beef and lamb offals	Boiled beef tripe, boiled beef feet, braised beef liver, boiled lamb brain, boiled lamb heart, boiled lamb kidneys, boiled lamb lungs, boiled lamb sweetbreads, boiled lamb spleen, grilled lamb liver, braised lamb liver
20-Chicken meat	Roasted chicken leg meat, fried chicken leg meat
21-Turkey meat	Roasted turkey breast, roasted turkey meat
22-Processed turkey meat	Cooked turkey salami
23-Olive oil	Virgin olive oil
24-Soybean Oil	Refined soybean oil
25-Bread, wholemeal	Wholemeal bread
26-Couscous, pasta, semoulina	Cornstarch, cooked pasta, couscous, cooked pearled barley, dried pearled barley, cooked semolina, dry whole grain wheat
27-Rice	Rice
28-Cereal flours	Cooked sorghum flour, cooked whole barley flour, cooked hard red wheat flour
29- Dry legumes	Boiled mature broad bean, cooked mature chickpeas, dried mature chickpeas (powder), cooked mature kidney beans, boiled lentils
30-Green legumes	Cooked fava, cooked broad bean (immature), cooked green peas, cooked green beans
31-Boiled potatoes	Boiled potatoes
32-Fried potatoes	French fried potatoes
33-Processed vegetables	Canned tomato paste
	Canned cooked mixed vegetables
34-Green-yellow vegetables	Broiled tomato, cooked carrots, cooked celery, cooked fennel leaf, fresh parsley, boiled tomato, raw tomato, fried tomato, fried green pepper, boiled green pepper, boiled pumpkin, lettuce leaves, cooked *Chorocus glinopus*, cooked chard, boiled whole beets
35-Other fresh vegetables	Cooked cardoon, raw cucumber, boiled turnips, boiled white onion, raw white onions
36-Salt	Added salt (iodized)
37-Spices	Black pepper, cinnamon powder, coriander seed, cumin seed, curry powder, fennel seed, ground thyme, dried spearmint leaves
38-Other condiments	Capers, cider vinegar, brewer's yeast, compressed baker's yeast,
39-Garlic	Fresh or cooked garlic
40-Harissa^2^	Home made harissa, mixed harissa, confectionnary harissa, red pepper powder
41-Tea	Tea
42-Coffee	Coffee
43-Water	Tap water

1-Chamia: typical oriental sweet preparation of sweetened sesame seeds paste often also including dried fruits or nuts.

2-Harissa: typical Tunisian very spicy chili preparation.

#### Computation of dietary pattern scores

The 134 food items of the food frequency questionnaire were grouped into 43 food groups (Table 1) based on empirical knowledge of Tunisian food habits and on the literature [35]. Intakes expressed in g/1000 kcal (i.e. g/4180 kJ) according to the nutrient density model [36] were recoded into quintiles prior to analysis. Data based dietary pattern scores [37,38] were then derived by multiple correspondence analysis (MCA): MCA is a multivariate exploratory data analysis technique closely related to principal component analysis (PCA), originally derived for categorical variables [39-43]. It can also be used to analyse categorised interval variables such as the food group variables in quintiles: it is then a robust non parametric method for extracting factors without any of the assumptions on which methods based on correlation coefficients depend. Similarly to PCA, MCA derives a series of orthogonal linear combinations of decreasing inertia. For each subject the score on a given axis is then a weighted linear combination of the binary variables coding her/his values on the quintiles of the 43 nutrient density variables. As in similar studies already published [44], choice was made to finally compute dietary patterns without stratification on gender for reasons pertaining to sample size and thus generalizability of the results, to preliminary analyses which suggested no major gender differences regarding inter-relationships between food groups variables, and also for comparability purposes when assessing associations with health outcomes.

The resulting scores were then used as interval variables after being rescaled from 0 to 100 for readability and also coded into tertiles for specific analyses. Characterisation of the dietary patterns was then as follows: - i) firstly, observed intakes of the 43 food groups were plotted against the different scores, - ii) secondly, intakes of selected macro and micro nutrients as well as DQI-I components were also plotted; to visualize overall trends both types of plot used local polynomial smoothing so as not make any a priori assumption on the linearity of associations and were done separately by gender to confirm that the interpretation of the scores was gender independent. Criterion validity was also indirectly assessed through associations of the dietary patterns with socio-economic variables.

#### Anthropometry

All anthropometric measurements were performed by personnel trained according to standard procedures: weight was measured to the nearest 100 g using electronic scales (Teraillon, France). Height was measured in a standing position, without shoes, to the nearest millimetre using portable gauges (Seca, Germany). Obesity was defined as BMI ≥ 95^th ^percentile and overweight (therefore including obesity) as BMI ≥ 85^th ^percentile based on the age and sex-specific BMI reference distributions [45,46]. Waist circumference (WC) was measured midway between the lower costal margin and the iliac crest to the nearest 0.1 cm with a non-elastic metric measuring tape with subjects standing in underclothes.

#### Blood pressure

Blood pressure was measured twice, first at the beginning (after 10 minutes rest), and second at the end of the interview, using calibrated sphygmomanometers (Vasquez Laubry type, Spengler, France). High blood pressure and hypertension were defined respectively: - for 15-17 y. as average systolic/diastolic BP ≥90th and respectively ≥95th percentile of the reference values for age, sex and height according to the standard definition introduced in 2003 and extended to children and adolescents in 2004 [47] - for 18-19 y. as average systolic/diastolic BP ≥120/80 mm Hg, respectively ≥140/90 mmHg.

### Data processing and analysis

Data entry screens including quality checks, as well as validation by double entry, used EpiData Software version 3.1 [48]. Data management and statistical analysis were performed using Stata 10 software [49]. Sampling weights were computed to account for differential probabilities of selection and also included a post-stratification on sex and urban *vs*. rural [50], and all analyses took into account stratification, clustering and sampling weights using the specific *svy *series of Stata commands. For multivariate analyses, the complete-case analysis approach was used to deal with missing data. The type I error risk was set at 0.05. Results are generally given as estimates ± design based standard error or also 0.95 confidence interval (in brackets) when relevant.

#### Analyses

Comparisons of food groups, nutrients, diet quality and physical activity data between genders adjusting for age, were performed using general linear models for interval response variables, or logistic regression for binary response variables.

**Figure 1:**
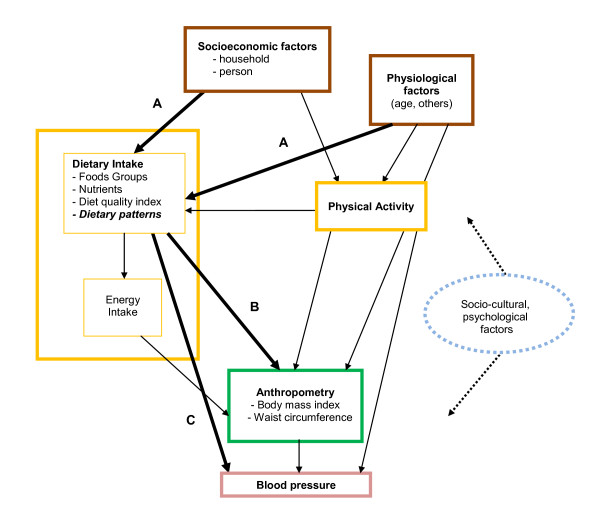
**Conceptual framework for analysis of associations between dietary patterns, socio-economic factors and health outcomes**. Conceptual framework for analysis of associations between dietary patterns, socio-economic factors and health outcomes. Arrows A, B and C (solid bold lines) represent the main associations studied, while the other associations represented (solid thin lines) were taken into account only to adjust for possible confounding; socio-cultural and psychological factors where not directly measured nor analysed in the study but represented so as to keep in mind that they may interact (dashed arrows) with the other factors at the different levels of the conceptual framework.

Assessment of the associations between socio-economic and physiological factors, dietary intake, and health outcomes was based on the conceptual framework presented in Figure 1[51]. Socio-economic factors were hypothesised to be the more distal, and their association with anthropometry or blood pressure to be mostly mediated through their association with the more proximal factors i.e. dietary intake and physical activity. Thus, first were studied the associations (arrow A) of socio-economic factors with the two diet pattern 0-100 variables using general linear models: univariate models were first fitted, then multivariate models to estimate adjusted associations. Then, separately for each gender, associations of diet scores (in tertiles) with anthropometry (arrow B ) were first estimated unadjusted (model 1), then adjusted for relevant confounders such as subject level physiological factors, as well as physical activity and mediating effect of total energy intake (model 2). In model 2, association of dietary patterns with blood pressure (arrow C) were, in addition, also adjusted for the possible mediating effect of anthropometry. We also tentatively tried to take into account residual confounding due to factors not directly assessed in the study e.g. cultural and/or psychological factors by adjusting for subject and household level socio-economic variables in a third type of models (model 3), despite this adjustment not being entirely consistent with the initial conceptual framework and the likely risk of over adjustment.

Models used were general linear models for interval response variables (BMI, WC, systolic and diastolic BP) to estimate crude and adjusted differences and logistic regression for binary response variables (overweight, high blood pressure and hypertension) to estimate crude and adjusted prevalence odds-ratios (POR).

### Ethics

This study was conducted according to the guidelines laid down in the Declaration of Helsinki and the protocol was reviewed and approved by the Tunisian Ministry of Health and the Tunisian National Council of Statistics (visa n°5/2005), and was conducted under the supervision of local medical authorities. The adolescents and their parents gave their verbal consent to take part in the study. The subjects were informed of their right to refuse to take part and of the strict respect of the confidentiality of their answers.

## Results

### General characteristics of the sample

From the 2820 selected households about 1235 subjects were to be included in the study (2004 national census): absence, refusal or missing or outlying dietary data resulted in 1019 subjects being analyzed. The weighted estimate of the proportion of females was close to half (Table 2) but with a lower response rate for males. Estimated percentages for socio-economic variables were similar between genders. Two thirds of the subjects were from urban households. A third was not registered at school.

**Table 2:** Socio-economic characteristics of the subjects

	All		Male		Female	
	n	**%**^**1**^	n	**%**^**1**^	n	**%**^**1**^
	
Physiological factors						
**Sex**	1019					
Male	432	50.3				
Female	587	49.7				
**Age (years)**	1019					
15 y.	225	22.5	91	21.3	134	23.8
16 y.	211	19.8	101	22.1	110	17.5
17 y.	204	20.1	84	18.8	120	21.4
18 y.	242	22.8	90	21.4	152	24.1
19 y.	137	14.8	66	16.4	71	13.2
**Environment**						

**Region**	1019					
District of Tunis	228	35.1	100	35.9	128	34.2
Middle Eastern	376	39.5	161	38.6	215	40.4
Middle Western	415	25.4	171	25.5	244	25.4
**Milieu**	1019					
Urban	566	67.4	246	67.6	320	67.2
Rural	453	32.6	186	32.4	267	32.8
**Socio-economic factors**						

**Economic level of the household**	972					
Upper tercile	248	33.6	182	36.2	231	33.2
Intermediate tercile	311	31.6	115	27.8	196	35.5
Lower tercile	413	34.8	119	35.9	129	31.2
**Profession of head of household**	1004					
Upper/intermediate	271	28.7	120	31.1	151	26.3
Employee/worker	590	56.0	340	52.5	350	59.5
Not working/Retired	143	15.3	65	16.4	78	14.2
**Education of head of household**	1007					
Secondary/University	317	37.1	133	36.3	184	38.0
Primary school or none	190	62.9	292	63.7	398	62.0
**Mother working outside the home**	1015					
Yes	131	14.9	56	13.7	75	16.2
No	884	85.1	375	83.6	509	83.8
**Education of mother**	1005					
Secondary/University	168	21.9	74	22.6	94	21.2
Primary school or none	837	78.1	350	77.4	487	78.8
**Currently attending school**	1015					
Yes	683	70.8	295	70.2	388	71.4
No	332	29.2	136	29.8	196	28.6

1- Weighted percentages.

### Food consumption and physical activity

Overall, the main constituents of the diet (detailed data not shown) in g/1000 kcal were legumes and vegetables (167.6 ± 4.0), white bread (95.8 ± 2.7), grains (70.8 ± 1.9), dairy products (63.1 ± 2.1) with also sizable quantities of fruits (36.5 ± 1.6) and sugars and confectionery (34.8 ± 1.2). Males reported consuming somewhat more dairy products, more sweets and less grain per 1000 kcal, than females, while consumption of fruits and vegetables was not sex specific (detailed data not shown). Overall energy intake (Table 3) was rather high (3341 ± 35 kcal or 13980 ± 145 kJ) when compared to usual intakes in industrialised countries and more so when expressed in percentage of physical expenditures (135.7% ± 1.8); energy intake was higher in males than in females (3442 ± 51 kcal vs. 3239 ± 40 kcal P = 0.001) but the percentage of expenditures was lower (121.1% vs. 150.5% P < 0.0001). The ratio of energy intake to body weight (57.9 ± 0.8 kcal/kg or 242.1 ± 3.3 kJ/kg) was similar in males and females and there was no sex difference in the overall structure of macronutrient intake. On average, Tunisian adolescent intakes of proteins, carbohydrates and fat, respectively 12, 52 and 36 percent of energy were not far from recommended levels [52]. Saturated fat (SAFA) represented 9%, mono unsaturated fat (MUFA) 14% and poly unsaturated fat (PUFA) 11% of energy intake. Average absolute dietary fibre intake was 36 g/day, above the recommended level (>25 g/day), with no difference between sexes per 1000 kcal. Total intake of free sugar, though similar per 1000 kcal, differed by sex, and was slightly above recommended levels (<10% of energy). Vitamin C and potassium intakes were well above the minimum recommended intake due to the high fruit and vegetable content of the diet, while average calcium intake was only two-thirds of the 1,300 mg/day required at these ages [53]. Iron intake on average was high, with bioavailability depending on the food source. For 1000 kcal, vitamin C intake was lower and calcium intake was higher for males than for females, while iron, zinc, sodium, potassium, folates and vitamin B12 intakes were similar.

**Table 3:** Nutrients, diet quality and physical activity data overall and by gender

	Overall (n = 1019)		Male (n = 432)		Female (n = 587)		
	**Mean**^**1**^	**s.e**.	**Mean**^**1**^	**s.e**.	**Mean**^**1**^	**s.e**.	**P-value**^**2**^
	
Dietary intake							
**Energy**							
Energy intake (kJ)	13980	145	14401	214	13767	168	0.001
Energy intake (kcal)	3341	35	3442	51	3239	40	0.001
Energy intake (% of expenditures)	135.7	1.8	121.1	2.3	150.5	2.3	<0.0001
**Macronutrients (g/1000 kcal)**							
Protein	31.3	0.2	31.2	0.2	31.3	0.2	0.55
Carbohydrates	130.5	0.6	130.4	0.8	130.7	0.7	0.69
Free sugar	26.8	0.4	27.2	0.5	26.4	0.5	0.30
Dietary fiber	10.8	0.1	10.7	0.1	10.8	0.1	0.49
Total fat	40.7	0.3	40.8	0.4	40.6	0.4	0.51
Saturated fat	9.5	0.1	9.5	0.1	9.4	0.1	0.52
Monounsaturated fat	15.5	0.2	15.6	0.3	15.3	0.3	0.43
Polyunsaturated fat	12.1	0.3	12.0	0.4	12.2	0.4	0.56
**Micronutrients (/1000 kcal)**							
Vitamin C (mg)	47.4	1.0	46.4	1.1	48.5	1.1	0.042
Calcium (mg)	265.9	3.1	271.2	4.2	260.5	3.5	0.027
Iron (mg)	6.7	0.0	6.7	0.0	6.7	0.0	0.55
Zinc (mg)	3.5	0.0	3.5	0.0	3.0	0.0	0.69
Sodium (mg)	1648.9	9.9	1641.5	13.3	1656.5	10.2	0.24
Potassium (mg)	1049.1	11.9	1044.5	14.9	1053.8	12.7	0.52
Folates (μg)	179.5	1.8	180.1	2.3	179.0	1.9	0.64
Vitamin B12 (μg)	2.4	0.1	2.4	0.1	2.4	0.1	0.96
**Diet Quality Index (DQI-I)**							
Total score (/100)	57.7	0.3	57.8	0.4	57.6	0.3	0.65
DQI-I ≥60	38.0%	2.1	39.7%	2.9	36.3%	2.5	0.37
Variety (/20)	13.8	0.2	14.1	0.3	13.4	0.2	0.022
Adequacy(/40)	33.7	0.1	34.0	0.2	33.4	0.1	0.0077
Moderation(/30)	9.0	0.2	8.5	0.3	9.4	0.3	0.018
Balance(/10)	1.3	0.1	1.2	0.1	1.3	0.1	0.55
							
**Physical activity level**							

Low intensity daytime activities (<3 BMR) (%)	83.4	0.6	78.8	0.9	87.9	0.4	<0.0001
Medium intensity daytime activities (3-6 BMR) (%)	15.1	0.6	18.7	0.8	11.5	0.4	<0.0001
High intensity daytime activities (≥6 BMR) (%)	1.5	0.1	2.5	0.1	0.6	0.1	<0.0001
Mean intensity of total daytime activity (BMR multiple)	1.7	0.1	1.8	0.0	1.6	0.1	<0.0001

1- Except for DQI-I ≥60 (weighted proportion of subjects with DQI-I  ≥60)

2- Comparison between sexes adjusted for age.

More than a third of the subjects were estimated as having a good quality diet (DQI-I > = 60). Scores of variety and adequacy were quite high, while components scores for moderation and overall balance were much lower. There was no difference between the sexes for global diet quality due to higher variety and adequacy for males, but a better moderation score for females.

The level of physical activity (Table 3) differed significantly between genders; on average males were more physically active, with more bouts of high and moderate intensity activities than females, who spent most of their time in a sedentary mode.

### Dietary patterns

**Figure 2:**
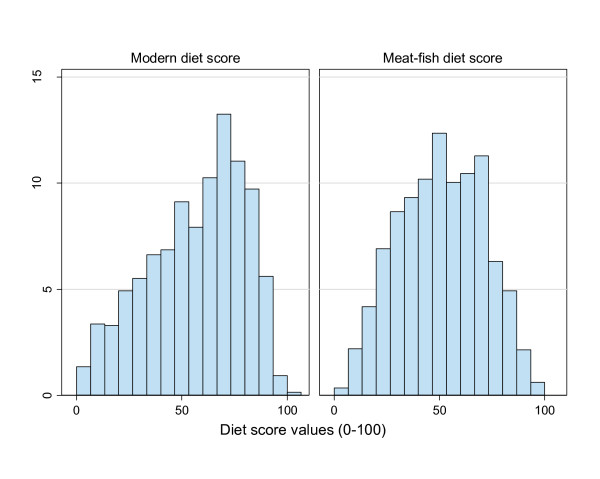
**Histograms of distribution of modern and meat-fish dietary pattern scores (n = 1019)**. Distribution of modern and meat-fish dietary patterns scores derived by multiple correspondence analysis of dietary intakes of the 43 food groups (coded in quintiles). The modern and meat-fish pattern are respectively the first and second principal axes. For each subject the score for a given pattern is a weighted linear combination of the binary variables coding her/his values regarding the quintiles of the 43 food groups. Each axis was rescaled from 0 to 100 for readability purposes.

Multivariate analysis of the 43 nutrient density food group variables resulted in retaining two dietary pattern scores (Figure 2) based on: - i) the scree test on the relative percentage of total inertia (axis 1 and 2 respectively 6.8% and 4.2%); also taking into account that for only technical reasons, relative % of inertia of MCA axes are usually lower than would be expected from other factorial methods such as PCA (due to the higher dimensionality of the data resulting from the binary coding of quintile data), - ii) interpretability of the axes as assessed by the association with the 43 food groups, nutrient data (g/1000 kcal) and DQI-I global and component scores.

#### Modern dietary pattern score

**Figure 3:**
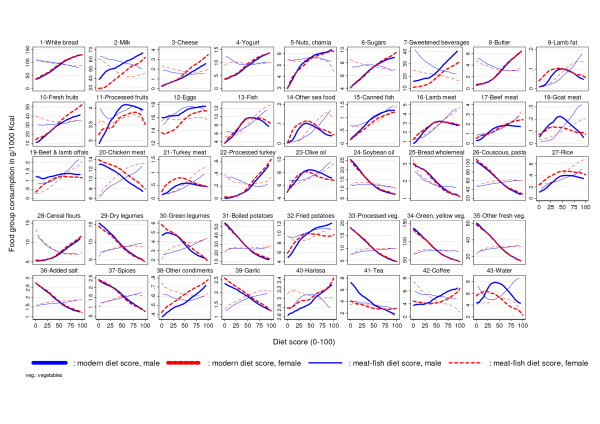
**Intakes of the 43 food groups according to the modern and meat-fish patterns**. By gender (male n = 432, female n = 587), according to the modern and meat-fish diet score patterns, intakes of the 43 food groups (in g/1000 kcal or g/4180 kj) from which the patterns were derived by multivariate analysis. Overall trends are estimated using local polynomial smoothing.

With reference to the food based observed associations (Figure 3), the first axis was interpreted in the Tunisian context as a gradient from a more "traditional" to a more "modern" food consumption pattern (from the lowest (0) to the highest (100) values of the score); hereafter it is thus referred to as "modern" dietary pattern.

*Food groups *: indeed, along the modern axis (Figure 3), overall consumption in g/1000 kcal, increased markedly for white bread, dairy products (major increases in milk and yogurt consumption, milder for cheese), sugars (major increase in sweet beverages), added fats (mostly from butter), fresh fruits, less so for eggs. On the contrary along this axis, consumption decreased for oils (sharp decrease in consumption of soybean oil), cereals and grains (remarkable drop in consumption of couscous and pasta not balanced by the mild increase for cereal flours); it decreased sharply for legumes and drastically for vegetables (marked reduction in consumption of boiled potatoes, of transformed and of both categories of fresh vegetables combined with a mild increase in consumption of fried potatoes). The relationship with meat, and notably fish, was usually not monotonous but with a small increase in consumption of canned fish and processed turkey as well as a decrease in consumption of chicken meat along the axis. Consumption of spices, added salt and garlic decreased along the axis, while that of harissa (very spicy short sauce) increased somewhat. Consumption of tea decreased while that of coffee increased along the axis. Overall, there were small sex specific deviations from this overall trend: along the axis there was a slightly larger increase for girls vs. boys for milk, for fresh fruits and eggs, while on the contrary, the increase in consumption of fried potatoes was a little less for girls.

**Figure 4:**
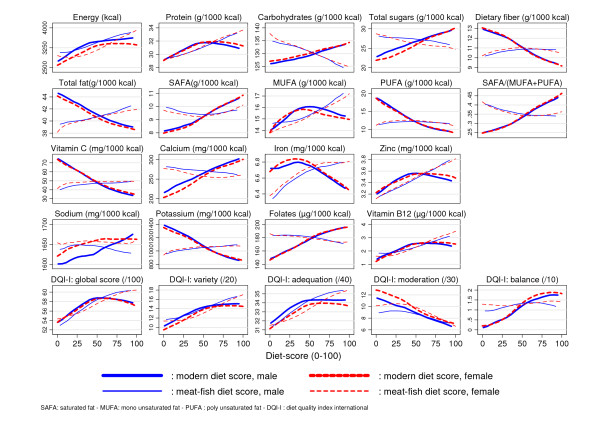
**Selected macro and micronutrient intakes and DQI-I scores according to the modern and meat-fish patterns**. By gender (male n = 432, female n = 587), intakes of selected macro and micronutrient and DQI-I (Diet Quality Index International) global and components scores according to the modern and meat-fish dietary patterns derived by multivariate analysis of the 43 food groups variables. Overall trends are estimated using local polynomial smoothing.

*Nutrients *: there was an increase along the axis in daily total energy intake in kcal but levelling off somewhat at mid-range (Figure 4). Protein intake increased somewhat, although the highest consumption was observed in the middle of the axis as the relationship levelled off after mid-range. Consumption of carbohydrates and of free sugar increased markedly while that of fibre decreased. Total fat and PUFA decreased sharply from one end of the axis to the other while SAFA increased; relationship with MUFA was not monotonous, increasing up to mid-range then decreasing somewhat. Vitamin C and potassium decreased markedly, the decrease for iron was milder; calcium increased sharply along the axis while there was a mild overall increase for sodium. Zinc and B12 increased moderately, but folate increased sharply all over the range of the score. As observed for food groups, there were few sex differences around these trends: only for sodium could there be observed a rather monotonous increase for boys, while for girls the curve was flatter and levelling from the mid-values of the axis.

*Diet quality *: A non linear relationship was observed with the DQI-I diet quality global score, with a sharp increase up to the mid-values of the axis, and then a slight decrease (with analogous results from prevalence of DQI-I ≥60, detailed data not shown): this stemmed both from the leveling of the increase in the variety, adequacy and balance components from the middle values of the diet score and the association with moderation featuring a clear linear decreasing trend.

As for dietary patterns derived by either PCA of Factor Analysis [38], interpretation must take into account that it is effectively a continuous gradient (Figure 2, 3 &4) of increasing modernisation of the diet from the left to the right of the x-axis, that could be also interpreted conversely as a measure of "traditionality" of the diet (from the right to the left of the x-axis).

#### Meat-fish dietary pattern score

*Food groups: *as for relationships between the second axis and the 43 food groups (Figure 3), the more straightforward observed associations were increasing linear gradients for fish and meat consumption. Decreasing associations with consumption e.g. of white bread, dairy products, sugars and confectionery and butter were also observed along the second axis but with differences much smaller than those observed along the first axis. No straightforward associations were observed for other food groups, except for an increase in consumption of olive oil.

*Nutrients *: total energy intake (Figure 4) increased very markedly and linearly along the axis; there was also a linear increasing relationship with total fat, MUFA, proteins and iron. As could be expected, zinc and B12, increased linearly with meat and fish intake, while folate did not change.

*Diet quality: *a salient feature of this second diet score was that due to a monotonous increase of the variety and adequation component scores (Figure 4), the global DQI-I score increased continuously along the whole range of axis values, with no flattening of the relationship as observed for the "modern" diet score; an analogous increase for prevalence of DQI-I≥60 was observed along the axis (data not shown).

Relationships of food groups or nutrient variables with this dietary pattern were mostly not sex specific. In the following, with reference to observed associations with the 43 food groups, this axis was termed as "meat-fish" diet pattern.

### Association of dietary patterns with socio-economic factors

*Modern diet score: *in unadjusted associations (detailed data not shown) males scored marginally higher (p = 0.04) than females on the modern diet score. Major differences were observed between regions and also urban vs. rural (+26.2 [21.1-31.4], P < 0.0001). The higher the economic level of the household, the more modern the diet (upper vs. low +33.8 [29.4-38.2], middle vs. low +19.2 [14.3-24.2], P < 0.0001). Upper/intermediate profession vs. not working (+6.0 [0.7-11.3], P < 0.0001), but most markedly a higher level of education (+17.4 [13.4-21.4], P < 0.0001) of the father, the mother working outside the home vs. not (+14.0 [9.7-18.2], P < 0.0001) and higher level of education of the mother (+22.1 [18.2-26.0], P < 0.0001) also resulted in the subjects having a higher mean value on the modern diet score. Those registered at school (vs. not) also scored higher (+14.7 [10.4-19.0]). In adjusted analyses, the slight association with sex persisted to a certain extent. Regional differences were still strong (Tunis vs. Center-West +27.8 [23.2-32.3], Center-East vs. Center-West +14.4[10.1-18.7], P < 0.0001) but the urban vs. rural contrast was reduced to only +4.8[1.1-8.5] (P = 0.012). A strong gradient of "modernisation" of the diet with increasing economic level of the household was still observed (upper vs. low +14.9 [10.8-19.0], middle vs. low +9.6 [6.2-12.9], P < 0.0001). The characteristics of the father were no longer associated, but there remained a mild independent trend towards a more modern diet among adolescents whose mother worked outside the home (P = 0.043), had a higher education level (P = 0.0013) and those registered at school (P = 0.030).

*Meat-fish diet score: *in unadjusted associations no differences were observed between the sexes, and only marginal differences for age, with older subjects scoring somewhat higher. Only the Middle Eastern Region (vs. Middle Western) scored higher (+15.0 [10.3-19.7]) and there was no urban vs. rural difference. As for the economic level of the household, the main difference was in the intermediate category (vs. lower) (+6.5 [2.6-10.3]). The only other marginally significant difference was observed among adolescents whose mother worked outside the home (-3.5 [-7.3-0.3]). Factors related to the father were not associated with the meat-fish diet score. After adjustment, there was still no independent association with sex and the older subjects scored slightly higher. The sharp contrast between the Middle Eastern and the Middle Western Regions remained unchanged, as did the milder one between the middle and low economic level. A mother working outside the home with a higher education level resulted in somewhat lower scores, while attending school (vs. not) was associated with a higher meat-fish score.

### Association of dietary intake with anthropometry and blood pressure

*Anthropometry*: the nutritional status of adolescents in the study area featured a mean BMI of 21.4 ± 0.1 kg/m^2^, (male 20.8 ± 0.2 vs. female 22.0 ± 0.2, P < 0.0001), the prevalence of overweight was 18.9% ± 1.5 (male 17.2% ± 2.3 vs. female 20.7% ± 2.1, P = 0.27) and obesity 4.3% ± 1.0 (male 4.0% ± 1.4 vs. female 4.6% ± 1.2, P = 0.78). Mean WC was 74.2 ± 0.4 cm (male 75.8 ± 0.6 vs. female 72.6 ± 0.4, P < 0.0001).

For both genders, no association was observed between tertiles of energy intake and BMI nor overweight, while for abdominal fat accumulation, only for boys was there a monotonous increase in mean WC with tertiles of energy intake, whether raw (P = 0.001) or adjusted (P = 0.021), (detailed data not shown).

In males, associations between the modern dietary pattern score and anthropometric measures of total and abdominal fat accumulation showed that BMI, prevalence of overweight and WC increased with modernisation of the diet (Table 4), though this relationship was not linear but levelled off between the 2^nd ^and 3^rd ^tertiles (e.g. % of overweight 2^nd ^vs. 1^st ^POR = 4.2[1.9-9.3], 3^rd ^vs. 1^st ^POR = 3.6[1.4-8.9]); these associations were minimally confounded by age, total energy intake or physical activity measures (e.g. overweight 2^nd ^vs. 1^st ^POR = 4.0[1.7-9.3], 3^rd ^vs. 1^st ^POR = 3.3 [1.3-8.7]). Adjustment for socio-economic factors changed the estimates somewhat more (e.g. % of overweight 2^nd ^vs. 1^st ^POR = 3.4 [1.5-7.9], 3^rd ^vs. 1^st ^POR = 2.3 [0.8-6.9]). In females, the only association observed with anthropometry, was with abdominal fat accumulation, those in the 3^rd ^vs. 1^st ^tertile of the modern score featuring a somewhat higher mean WC in unadjusted analysis, but the strength of the association was further reduced in adjusted analyses. Regarding the meat-fish dietary pattern score, a higher mean BMI and WC was observed in males in the 3^rd ^tertile, either adjusted of not. No obvious relationship was observed in females.

**Table 4:** Association of dietary pattern scores with anthropometry and blood pressure data

	Modern dietary pattern score						Meat-fish dietary pattern score					
	
	Mean (s.e.) or %			P-value^1^			Mean (s.e.) or %			P-value^1^		
	
	1^st ^tertile	2^nd ^tertile	3^rd ^tertile	Model 1	Model 2	Model 3	1^st ^tertile	2^nd ^tertile	3^rd ^tertile	Model 1	Model 2	Model 3
	
Male	n = 170	n = 133	n = 94				n = 127	n = 143	n = 127			
Body Mass Index (kg/m^2^)	19.7(0.2)	21.7(0.3)	20.7(0.4)	<0.0001	<0.0001	<0.0001	20.1(0.3)	20.3(0.3)	21.6(0.4)	0.0035	0.092	0.059
Overweight (%)	6.7%	23.0%	20.4%	0.0026	0.0051	0.014	16.0%	11.0%	23.4%	0.063	0.23	0.31
Waist Circumference (cm)	71.0(0.6)	78.2(1.1)	77.1(0.8)	<0.0001	<0.0001	<0.0001	71.0(0.6)	78.2(1.1)	77.1(0.8)	<0.0001	<0.0001	<0.0001
Systolic blood pressure (mm hg)	113.9(0.9)	116.4(1.2)	112.7(1.2)	0.11	0.13	0.10	113.0(0.8)	113.9(1.1)	116.1(1.2)	0.098	0.40	0.43
Diastolic blood pressure (mm hg)	66.0(0.8)	69.1(0.7)	68.4(0.9)	0.022	0.044	0.073	66.7(0.8)	67.6(0.7)	69.1(0.7)	0.045	0.47	0.36
High blood pressure (%)	43.2%	53.0%	40.7%	0.15	0.28	0.23	44.3%	42.4%	50.5%	0.47	0.65	0.40
Hypertension (%)	3.1%	7.8%	2.9%	0.25	0.30	0.038	1.4%	4.8%	7.7%	0.11	0.16	0.15
**Female**	n = 263	n = 165	n = 108				n = 193	n = 167	n = 176			

Body Mass Index (kg/m^2^)	21.9(0.2)	21.7(0.3)	22.1(0.4)	0.77	0.65	0.68	22.2(0.3)	21.4(0.3)	22.1(0.3)	0.078	0.056	0.084
Overweight (%)	16.6%	20.2%	21.8%	0.58	0.51	0.41	20.7%	18.0%	19.2%	0.83	0.81	0.96
Waist Circumference (cm)	71.2(0.6)	72.7(0.9)	73.2(0.8)	0.083	0.10	0.15	71.7(0.7)	71.6(0.7)	73.3(0.7)	0.13	0.18	0.21
Systolic blood pressure (mm Hg)	113.3(0.7)	110.6(0.9)	108.9(1.0)	0.0028	0.0011	0.22	110.9(0.9)	111.8(0.8)	110.9(0.9)	0.64	0.50	0.57
Diastolic blood pressure (mm Hg)	66.1(0.7)	66.8(0.9)	66.2(0.8)	0.77	0.95	0.30	65.6(0.8)	66.5(0.7)	67.0(0.8)	0.43	0.92	0.87
High blood pressure (%)	39.3%	26.9%	21.4%	0.028	0.0085	0.31	32.2%	27.8%	30.0%	0.70	0.35	0.32
Hypertension (%)	7.0%	7.6%	0.0%	0.0041^2^	^2^	^2^	6.3%	3.0%	6.2%	0.44	0.38	0.38

1- Null hypothesis of no difference between tertiles of dietary pattern: general linear model for interval response variables, logistic regression for binary variables (complete case analysis n = 933).

Model 1: unadjusted estimates (response variable as in column 1, regressor is categorical variable for dietary pattern in tertiles).

Model 2: for BMI, overweight and WC response variables: adjusted for age, total energy intake, physical activity level (% of low, medium and high intensity daytime activities); for blood pressure (systolic and diastolic), high blood pressure and hypertension response variables: adjusted also for anthropometry (BMI and WC).

Model 3: as in model 2 but in addition adjusted also for socio-economic variables: economic level of household, profession of father, education of father, mother working outside the home, education of mother, whether or not subject is attending school at time of survey.

2- For females, P-value for unadjusted association is from chi-square test (also taking into account sampling design), as no estimates could be obtained for logistic regression models due to observed null prevalence in third tertile (and thus no raw and/or adjusted p-values).

*Blood pressure: *overall mean systolic BP (mm Hg) was 112.8 ± 0.4 (male 114.4 ± 0.6 vs. female 111.1 ± 0.5, P < 0.0001), and diastolic 67.2 ± 0.4 (male 67.9 ± 0.5 vs. female 66.4 ± 0.5, P = 0.020). Prevalence of high blood pressure was 37.4% ± 1.8 (male 44.74% ± 2.7 vs. female 30.0%±2.3, P = 0.0001) and that of hypertension 5.2%±1.0 (male 5.0%±1.4 vs. female 5.3%±1.0, P = 0.96).

As for associations between levels of energy intake (in tertiles) and BP (detailed data not shown), none were observed for boys. Among females after adjustment there was an increase in levels of high blood pressure with tertiles energy intake (2^nd ^vs. 1^st ^tertile POR = 2.1[1.2-3.5], 3^rd ^vs. 1^st ^tertile POR = 1.7[1.0-2.9]).

As for association with dietary patterns: - i) in males, except for the mild bell-shaped association with diastolic blood pressure and hypertension, no straightforward associations were observed between BP and the modern diet score. For the meat-fish diet score, although before adjustment for energy intake, BMI and WC, there was an increase in diastolic blood pressure, no major associations were observed either (Table 4). - ii) for females, due to the decrease in systolic BP along the modern dietary pattern score, there was a decreasing gradient in the prevalence of high blood pressure with modernisation of the diet: (2^nd ^vs. 1^st ^tertile POR = 0.6 [0.3-1.0], 3^rd ^vs. 1^st ^tertile POR = 0.4 [0.2-0.8]); this association was minimally reduced by adjustment for age, total energy intake or physical activity measures (2^nd ^vs. 1^st ^tertile POR = 0.5 [0.3-0.8], 3^rd ^vs. 1^st ^tertile POR = 0.4 [0.2-0.8]) but confidence intervals were widened by adjustment on socio-economic factors (2^nd ^vs 1^st ^tertile POR = 0.6[0.3-1.2], 3^rd ^vs 1^st ^tertile POR = 0.6[0.3-1.5]). Was also observed a lower prevalence of hypertension in the highest tertile of the "modern" dietary pattern (though for computational reasons due to the observed null prevalence, it was not possible to obtain estimates of the PORs either crude or adjusted).

## Discussion

The present study aimed at assessing dietary intake and patterns, as well as associations with socio-economic cofactors and health outcomes such as overall nutritional status and blood pressure, among Tunisian adolescents.

As for overall energy intake, within the limits of the estimation of dietary intake using a semi-quantitative food frequency questionnaire, we found high overall intake by both males and females, largely exceeding the requirements based on their physical expenditure level, and more so in females than in males. Indeed, we found close average energy intake levels between boys and girls, contrary to what has been observed over the last decades in Europe, where, whatever the methodology employed, females always displayed much lower energy intakes than males [54]. In this context, changes in energy expenditure for girls vs. boys were also accompanied by a modification of the desirable body image for girls which resulted in an even more drastic decrease in energy intake over past decades than for boys [54,55]. Obviously, this change is not yet fully at work in Tunisia where girls still maintain, relative to boys, a high level of food energy consumption despite a quite sedentary lifestyle; also, it has been shown that if there is indeed a shift from the cultural attitude favouring plumpness towards preference for a normal corpulence, younger women do not systematically value slimness even in urban settings [56]. Although it is difficult to derive longitudinal inferences from cross sectional data especially given the rapid economic development and societal changes in Tunisia in the last 30 years, these observations are coherent with obesity in Tunisia being much more prevalent among adult women vs. men [13,17] while obesity figures are much more gender independent in European countries.

Though not far from recommendations, on average the diet was a little high in fat as a % of energy, as is common in European Mediterranean countries, with a favourable ratio of unsaturated fats due to the consumption of olive oil [52]. As the consumption of fruit and vegetables continued at a rather high level, well above the recommended threshold of 400 g/day [52], the fibre and micronutrient contents of the overall diet appear to be relatively satisfactory with the exception of calcium. However, only 38% of adolescents displayed a satisfactory diet quality score (DQI-I), generally due to a good level of variety and adequacy but a low score for moderation and balance.

Concerning food based descriptive analysis of dietary intake by computation of dietary patterns, the main dimension underlying food consumption data was found to be a traditional to modern gradient, characterised from the food point of view by a linear decrease in consumption of long-established foods such as vegetable oil, cereals, grains, legumes and vegetables and an increase in foods made more widely available in the last decades such as white bread, dairy products, sugars and confectionery, added fats, fried potatoes and fruits. It is of course always difficult from a methodological standpoint to discuss longitudinal inferences based on cross sectional data. But anyway, this finding is in line with a chronological food transition process [57] that would be actively underway as strong unconfounded associations were observed with environmental and socio-economic cofactors: indeed, in our study the diet was all the more 'modernised' in urban or the more affluent households, or for a higher level of education of the mother, indicating that there was, at the time of the study among Tunisian adolescents a gradient of nutritional transition correlated to a gradient of socio-economic development within the country. It may be hypothesised that this dietary gradient is likely to recede if/when socio-economic development, lifestyle and dietary practices uniformise and give way, within a globally "modernised" food consumption, to different patterns such as those observed in studies among youth of industrialised countries [44,58,59], where such a gradient was obviously not observed. But as for now, from a descriptive point of view, this main traditional to modern gradient is a characteristic dimension underlying the variability of food based dietary intakes among Tunisian youth.

A second gradient, though of less importance, was observed, characterised by a monotonous and increasing relationship with energy, meat and fish consumption and somewhat decreasing with white bread, milk, sugars and sweet beverages and added fats. Interestingly, it revealed another path to a modified diet accompanying economic improvement, which yielded a better quality diet (DQI-I) for the last tertile than the modernised diet, with still a better ratio of unsaturated to saturated fats due to an increase in olive oil consumption, despite its richness in animal proteins; however, it also featured an excess of energy. It is apparently rather specific to the coastal Middle Eastern Region in Tunisia and to intermediate economic level households, without much independent effect of other socio-economic cofactors.

Even after adjustment (including for physical activity), with the exception of WC for boys, there was no straightforward relationship between anthropometry and energy intake or imbalance in neither gender. However, such a null association is rather frequently observed in either cross-sectional or even prospective studies. Underreporting, methodological errors, insufficient measurement precision or accuracy of energy intake and expenditure or dieting have successively been invoked [60,61]. On the other hand, there was a marked association between the modern diet score and both BMI and WC, but only in males. In Iranian adolescents a linear relationship between the consumption of high fat and salty snacks and carbohydrates (bread, rice pasta) and BMI was also observed, though total energy did not differ between overweight or obese and non-obese subjects [62]. Among females, even after adjustment for age, total energy intake, physical activity and anthropometry, modernisation of the diet was associated with a decrease of high blood pressure and hypertension (though somewhat levelling off after the 2^nd ^tertile). It is to be noted that the association was somewhat reduced from a statistical point of view by adjustment on socio-economic factors; nevertheless, it is debatable whether this third type of adjusted estimate is the relevant one for assessing association of diet patterns with health outcomes. Indeed, some authors do not adjust for socio-economic factors when assessing relationship between health outcomes and dietary patterns, which is coherent with a conceptual framework such as that presented in Figure 1, where these latter factors are the more distal ones, whose effect on health outcomes is hypothesised to be mostly mediated through the proximal ones i.e. dietary intake and physical activity. Our tentative adjustment on socio-economic factors in the third type of models was an attempt to try and take into account psychological and/or sociocultural differences that might be linked to socio-economic status in the Tunisian context, but some kind of over adjustment cannot be ruled out. From our first type of adjusted estimate (i.e. adjustment for age, total energy intake, physical activity and anthropometry only) it would seem that girls with a somewhat modernised diet have a reduced risk of arterial hypertension. In adults, it has been shown that diets high in potassium, vitamin C, or low fat dairy products, and low in sodium, were effective to reduce BP significantly [63-66]. However, previous studies have not revealed always consistent associations in adolescents between systolic BP and diet components [44,67]. Nevertheless, in northern Greece, decreased systolic BP was found to be associated with decreases in potassium and increase in calcium intakes among adolescents [68]. Although the evidence regarding associations between calcium intake and BP is somewhat mixed [69], other studies also mentioned the potentially significant role of calcium: dairy foods were an important component of the DASH (Dietary Approaches to Stop Hypertension) diet, and calcium supplementation has been shown to be associated with a modest reduction of BP, even in the absence of a decrease in sodium, specifically when calcium intakes were initially rather low [63,70-72]. Indeed, in the present study, the level of added salt decreased along the modern diet pattern axis, but not the total sodium intake, with however a slight difference between males and females. Potassium and vitamin C also decreased, while calcium rose significantly along with modern diet score tertiles. Zinc, B12 and in particular folates increased significantly with a modern diet, these nutrients being generally associated with a better blood pressure status, even in children [73].

Not so many studies have tried to distinguish dietary patterns in adolescents in relation with their health status in industrialised countries [44,74-78] and even fewer in emerging countries [79,80]: the study [79], though using cluster analysis instead of a method related to principal components or factor analysis, showed in Korea results comparable to those shown here, in that the transition to a modernised western-type diet had both positive and negative potential health consequences, improving the variety of the diet but increasing the level of fat or sugar for instance [79].

As for the strong and weak points of the study, regarding design and sampling, the cross-sectional design with retrospective estimates of exposure has known limitations when trying to interpret observed associations as causal. The study carried out in 2005 is quite recent but given the rapid pace of societal and socio-economic changes in emerging countries such as Tunisia it cannot be ruled out that further changes may have occurred since. The stratified, clustered random sample of 1019 subjects, although not national, was representative of three regions that feature a panel of the socio-economic and nutritional situations in Tunisia, as assessed by observed distributions of socio-economic factors and nutritional status indicators with reference to a national survey [23]; there was nevertheless a somewhat lower response rate for males vs. females which is not unusual in this context; we used weighting factors to partly control for non response but this does not preclude some amount of selection bias.

Although widely used in other fields, the MCA technique itself has previously been rarely used for computation of dietary patterns compared to PCA or factor analysis, or only with dichotomous coding of food group intakes [40,42]. In our study, use of MCA applied to food groups intakes in quintiles enabled a non parametric and robust assessment of associations between consumptions of the 43 food groups and deriving dietary patterns without relying on the assumptions on which methods based on correlation coefficients depend and which rarely hold in data from large food consumption surveys. Moreover, one common advantage of using methods related to PCA (i.e. PCA itself but also factor analysis and MCA), deriving patterns which are linear combination of the initial variables vs. methods related to cluster analysis, is that it results in scores, for which a dose-effect relationship with health outcomes can be assessed. We could also have tried to derive dietary pattern predictive of the studied health outcomes [37,81], but did not do so as our main objective was to assess dietary practices among Tunisian adolescents from a descriptive point of view.

Regarding measurements, assessment of dietary intake by a retrospective frequency questionnaire does raise a number of concerns [82] especially accuracy of estimates of subject level absolute intakes in energy and other nutrients, although at population level individual variation might be mitigated; also these drawbacks are likely less of an issue regarding computations of food list based diet scores. Although memory bias is known to increase with the length of the recall period, the one month retrospective period was an advantage regarding estimates of usual intakes (vs. instruments measuring either prospective or retrospective intakes over shorter periods); also the questionnaire was validated and adapted to the local context and - contrary to some other studies- included portion size data. Some seasonal difference probably affected availability of some foods during the survey period, although the most important seasonal change is between the winter season and the rest of the year. Regarding assessment of diet quality, some authors do not approve of the universal use of the DQI-I based on U.S. guidelines, preferring to derive specific indices, e.g. in West Africa based on WHO and FAO recommendations [83,84]; nevertheless which of those indices should be used among those already proposed is an open question as all those indices do have specific limitations and often fulfill different purposes [85].

A specific strength of the study was to feature, through a validated questionnaire, a direct and detailed assessment of physical activity and thus control of confounding related to this dimension.

Regarding assessment of overweight and obesity, there are, indeed, a number of issues regarding the choice of international references and/or the use of a national reference [86-88]. Our choice was made for comparability issues with previously published data on the whole country from which the subjects of the food consumption survey are a subsample [23], taking into account that descriptive anthropometric status data was not the main focus of the present study.

If the nutrient related hypotheses put forward to explain decrease of BP with modernisation of the diet among girls are coherent with the observed associations, some amount of confounding by factors not taken into account in the study cannot be ruled out. Indeed, the modernisation of the diet could be a correlate of a modernised lifestyle which, all the more for girls in the Tunisian context, could have both indirect effects through dietary intake and physical activity, but also, direct effects on measured health outcomes. It should then be of interest to assess dimensions such as health related quality of life, perceived stress or family psychosocial factors [89,90]. More research is likely needed on these issues which would likely be more appropriately dealt with by a mixed quantitative and qualitative approach.

## Conclusion

The present study is one of the few providing evidence regarding transition related dietary and health issues among adolescents from emerging North African or Middle East Arab countries, of which Tunisia is emblematic: indeed, although one must be careful not to overstate similarities between these countries, especially from a development point of view, their youth do share common consequences of globalization and related overall and rapid economic and societal changes as well as a number of common socio-cultural issues.

The analysis of the diet of these adolescents revealed a main pattern of modernisation along with urbanisation and regional socio-economic development leading to a more varied and adequate diet that prevents nutrient deficiencies. A favourable effect on BP in girls likely linked to more dairy consumption was also observed, although some amount of confusion by psycho-social factors cannot be entirely ruled out; nevertheless, this association points out that one should perhaps avoid systematically shedding a negative light on a modernised diet vs. an idealised traditional one. However, this modernisation gradient was also correlated with an excess of energy and nutrients such as saturated fat involving a higher risk of chronic diseases, while protective antioxidant micronutrients like vitamin C decreased notably.

The root causes of dietary changes and the nutrition transition are at a very macro level linked to global socio-economic and societal changes; nevertheless, interventions aimed at adolescents or their parents [91,92] to promote healthy lifestyles including limiting energy intake are likely part of the measures to be implemented to prevent the negative effect of the current food transition and its future impact on the burden of chronic diseases when this generation reaches adulthood.

## Competing interests

The authors declare that they have no competing interests.

## Authors' contributions

HAS designed the study, supervised data collection, contributed to data analysis and drafted the manuscript; PT planned and performed data analyses, contributed to interpretation of the results and to writing the manuscript; JEA contributed to the interpretation of the food consumption data and helped edit the manuscript; SED and EL helped with data management and interpretation of food consumption data; FD and NA contributed to the study design and revision of the manuscript; HBR and BM were involved in all steps, from the design of the study to the revision of the manuscript. All authors read and approved the manuscript.
